# Variables that influence the medical decision regarding Advance Directives and their impact on end-of-life care

**DOI:** 10.31744/einstein_journal/2020RW4852

**Published:** 2019-10-03

**Authors:** Larissa Mont’Alverne de Arruda, Kelline Paiva Bringel Abreu, Laryssa Braga Cavalcante Santana, Manuela Vasconcelos de Castro Sales

**Affiliations:** 1 Hospital Haroldo Juaçaba Instituto do Câncer do Ceará FortalezaCE Brazil Hospital Haroldo Juaçaba, Instituto do Câncer do Ceará, Fortaleza, CE, Brazil.; 2 Instituição Federal das Unimeds do Estado do Ceará FortalezaCE Brazil Instituição Federal das Unimeds do Estado do Ceará, Fortaleza, CE, Brazil.; 3 Hospital de Messejana Dr. Carlos Alberto Studart Gomes FortalezaCE Brazil Hospital de Messejana Dr. Carlos Alberto Studart Gomes, Fortaleza, CE, Brazil.; 4 Hospital Universitário Walter Cantídio FortalezaCE Brazil Hospital Universitário Walter Cantídio, Fortaleza, CE, Brazil.

**Keywords:** Advance directives, Living wills, Physicians, Attitude, Decision making, Advance care planning

## Abstract

The objective of this study was to identify the variables that influence physicians to implement Advance Directives and assess their impact on end-of-life care. It is a narrative literature review of 25 articles published between 1997 and 2018, in the following databases: CAPES, EBSCOhost, BDTD, VHL, Google Scholar, MEDLINE^®^/PubMed. The keywords utilized were: “advance directives”, “living wills”, “physicians”, “attitude”, “decision making”, “advance care planning”. The main factors that influenced physicians to implemente the directives were patients prognosis, medical paternalism, and patients understanding of their medical condition. Respect for autonomy, lack of knowledge and experience with directives, legal concerns, family influence, cultural and religious factors also contributed to medical decision. Most studies (86%) showed that having a directive led to lower rates of invasive interventions in the last days of patient´s life. Physicians were interested in respecting their patients’ autonomy and agreed that having an advance directive helped in the decision-making process; however, they stated other factors were also taken into account, mainly prognosis and reversibility conditions. Having directives contributed to reducing the use of life support therapies and adoption of comfort measures.

## INTRODUCTION

Continuous technological advances make professionals attempt to extend the life of their patients, regardless of the conditions they are living. In terminally ill patients, invasive support measures no longer increase their survival, but just prolong the process of death. Current medicine searches for the sensible balance in the doctor-patient relationship, aiming to guarantee the patient’s autonomy, including the principle of non-maleficence. Advance Directives (AD) have been gaining importance in defining the patient´s plan of care.^1 , 2^

Advance Directives are statements written in advance by patients, where they express their wishes and preferences of treatment, freely and autonomously, in the final moments of their existence, due to permanent illness or disability. The objective is to protect patients’ autonomy, shall they become unable to decide for themselves in the future.^3 - 6^

Some studies demonstrated that most critically ill patients lose their decision-making capacity, which will, more and more, in a complex way, be handled at the discretion of the family and attending physicians. Patients’ wishes regarding future procedures and treatments must be previously identified.^7 , 8^ Although most physicians demonstrate positive attitudes towards AD, the attending team does not always follow the plan of care established in the AD.^5 , 9 , 10^

In this study, we reviewed the literature to identify the variables that influence physicians to implement the AD, and to evaluate the impact of having an AD in the end-of-life care.

This study is a narrative review of the literature carried out between July and October 2018, in which the articles published in the previous 21 years (1997 to 2018) in English, Spanish, and Portuguese, in the databases of the Coordination of Improvement of Higher Education Personnel (CAPES), EBSCOhost, *Biblioteca Digital Brasileira de Teses e Dissertações* (BDTD), Virtual Health Library (VHL), Google Scholar, and MEDLINE^®^/PubMed were reviewed. The Health Science Descriptors used were “advance directives”; “living wills”; “physicians”; “attitude”; “decision making”; “advance care planning”, along with the Boolean operators AND/OR.

We considered all articles publication that identified any of the following factors: the physicians’ attitude towards the directives (positive, negative or mixed) with the variables in the decision process and/or the effects of having directives in the end-of-life care. Original articles (observational studies, cohort studies, clinical trials) and systematic reviews with quantitative, qualitative or mixed methodology were included. Theses, book chapters, case reports, publications in languages other than Portuguese, Spanish or English, studies that identified only the attitude of non-medical healthcare professionals, articles with emphasis only on the knowledge and experience of physicians regarding directives, as well as duplicate articles, were excluded.

We defined as directives the explicit manifestation of will or “living will” (LW), durable power of attorney for health care (DPA), both (LW and DPA), and “do not resuscitate” (DNR).

### Variables that influence the medical decision regarding Advance Directives

During the search process in the databases, 88 articles were initially selected by reading of abstracts. After detailed analysis of the inclusion and exclusion criteria, in addition to the articles found in duplicate (only two), 63 papers were excluded. Of the 25 studies included (1 systematic review and 24 original articles), the variables influencing physicians’ implementation of the directives were analyzed in 18 studies ( Table 1 ).

**Table 1:** Factors that influence the attitude of physicians towards Advanced Directives

Study	Autonomy	Knowledge and experience	Specific scenario	Paternalism and patients´ understanding	Legal issues	Family	Cultural and e religious factors
Rossini et al.^(1)^		+					
Quantitative							
Questionnaire							
Sittisombut et al.^(2)^							+
Quantitative							
Questionnaire							
Bradley et al.^(6)^				+			
Qualitative							
Interview							
Hildén et al.^(11)^	+						
Quantitative							
Questionnaire							
Torke et al.^(12)^	+		+				
Quantitative							
Questionnaire							
Thompson et al.^(13)^	+						
Qualitative							
Interview							
Navarro et al.^(14)^		+					
Quantitative							
Questionnaire							
Velasco-Sanz et al.^(15)^		+					
Quantitative							
Questionnaire							
Peicius et al.^(16)^		+					
Quantitative							
Questionnaire							
Burkle et al.^(17)^			+		+	+	
Quantitative							
Hypothetic scenarios							
Bond et al.^(18)^				+			
Qualitative							
Interview							
Bentur et al.^(19)^				+			+
Qualitative							
Interview							
Asai et al.^(20)^					+	+	+
Qualitative							
Interview							
White et al.^(21)^					+		
Quantitative							
Questionnaire							
Forte et al.^(22)^					+		
Quantitative							
Questionnaire							
Escher et al.^(23)^						+	
Quantitative							
Questionnaire							
Hypothetic scenarios							
Horn et al.^(24)^							+
Qualitative							
Interview							
Sprung et al.^(25)^							+
Prospective							

Based on the studies presented in table 1 , the main factors that influenced healthcare professionals in adopting the AD were respect for autonomy,^11 - 13^ lack of medical knowledge and experience in the use of directives,^1 , 14 - 16^ medical paternalism and difficulties in defining the patient’s prognosis,^6 , 17 - 19^ legal concerns,^17 , 20 - 22^ family influence,^17 , 20 , 23^ and cultural and religious factors.^2 , 19 , 20 , 24 , 25^ Respect for autonomy was demonstrated by Torke et al., where 96.6% and 81.8% of physicians considered “respect patient as a person” and “what patients wished would be done with them” as extremely or very important factors, respectively, in the medical decision process.^12^

The knowledge and experience of physicians in the use of directives are directly related to their greater use in decision-making process.^1 , 14 - 16^ Most agree that having directives and the formalization of the document help in the decision-making process. In a study with family physicians, approximately 70% were knowledgeable about the subject, but lack of deeper specific knowledge in order to counsel patients was one of the main barriers.^14^ Other studies conducted in countries where AD have not been legalized yet present much higher rates of unawareness, ranging from 50 to 74%.^1 , 16^

Burkle et al., have suggested that medical compliance to a directive is a “specific situation”, and medical judgment is more important than following an existing AD, depending on the patient’s clinical condition. The consonance would be greater in situations of chronic diseases, terminally ill patients or individual´s suffering, while in emergency and/or reversible situations, medical judgment would prevail.^17^

Paternalistic attitude from health professionals, that is, the decision of a medical intervention without considering the patient’s opinion aiming at their benefit, as well as patient’s not understanding the true situation of their illness also interfere in the medical decision-making process.^6 , 18 , 19^ Bond et al., demonstrated that the level of agreement with the directives is directly related to what physicians think is best for the patient, suggesting that if patients had all scientific knowledge, they would agree with the medical decision.^18^ Some studies suggested that patients are not correctly informed when creating a directive, in such a way that it may represent a misinterpretation of their desires.^6^

In countries where AD are legally protected, much is questioned about the legal influence on medical decision-making.^17 , 20 - 22^ In a survey conducted in three different Australian states, with their respective laws, most interviewees considered AD in the decision process, but other factors were more important, such as the patient’s quality of life after the proposed treatment and indication of clinical treatment, rather than the legal obligation of respecting a directive.^21^ Many physicians, however, admit that fear of legal accountability may affect their decisions regarding a patient. In hypothetical scenarios of disagreement between family opinions and an existing directive, 53% of interviewees considered legal concerns to be important or very important for their decision.^17^ Similarly, Forte et al *.,* found that 44% of physicians would change their behavior if it were not for the fear of lawsuits and the opinion of society.^22^

Family influences are also pointed out as reasons for the negative attitudes of physicians towards the patient’s choices.^17 , 20 , 23^ Burkle et al., found that 75% of physicians will honor patients’ directives, regardless of family opinion. However, among those who did not comply, the majority (77%) considered the family opinion an important factor for their decision.^17^

Other negative aspects in the implementation of AD are cultural and religious factors.^2 , 19 , 20 , 24 , 25^ Asian physicians tend to discuss the prognosis of their patients with relatives and, therefore, may not respect existing directives.^2 , 20^ Sittisombut et al., showed that 61.8% of Thai physicians never asked terminally ill patients if they wished to undergo cardiopulmonary resuscitation (CPR), however 94.5% discussed this issue with the family members.^2^ Sprung et al., found that Protestant, Catholic or non-religious physicians interrupted Advanced Life Support (ALS) more often than Orthodox Greeks, Jews or Muslims.^25^

### Impact of having Advance Directives in the end-of-life care

The impact of having end-of-life care directives was assessed in seven studies ( Table 2 ).^7 - 9 , 26 - 29^

**Table 2:** Influence of Advanced Directives on end-of-life care

Study	Type	Population	AD	Outcome	aOR (95%CI)
Hartog et al.^(7)^	Retrospective	192 ICU patients	Yes *versus* No	CPR	9.4 *versus* 22.8%*
			n=64 *versus* 128	DNR	76.6 *versus* 56.3%*
				MV	79.7 *versus* 89.7%
				Vasopressors	85.9 *versus* 87.9%
				Hemodialysis	42.2 *versus* 46.3%
Silveira et al.^(8)^	Retrospective	999 patients >60 years	LW *versus* No LW	Death at hospital	0.71 (0.47-1.07)
			n=444 *versus* 552	Full	0.33 (0.19-0.56)*
			DPA versus No DPA	Limited	1.79 (1.28-2.50)*
			n=589 *versus* 407	Comfort	2.59 (1.06-6.31)*
				Death at hospital	0.72 (0.55-0.93)*
				Full	0.54 (0.34-0.86)*
				Limited	1.18 (0.75-1.85)
				Comfort	2.01 (0.89-4.52)
Brinkman-Stoppelenburg et al.^(9)^	Systematic review	113 patients with cancer, advanced dementia, trauma or cardiopathy	n=45	ALS (n=22)	10/22 positive studies
			Yes *versus* No	Quality of life (n=6)	0/6 positive studies
				Length of hospital stay (n=8)	2/8 positive studies
				Palliative care (n=7)	5/7 positive studies
				Family/patient stress (n=8)	3/8 positive studies
				Respect to patient desires (n=2)	0/2 positive studies
Cappell et al.^(26)^	Retrospective	422 patients submitted to allogenic transplant	Yes *versus* No	Admission to ICU	41 *versus* 52%*
			n=184 *versus* 238	MV	21 *versus* 37%*
				Death at ICU	0.44 (0.27-0.72)*
Yen et al.^(27)^	Prospective	1,307 patients >65 years with chronic diseases	Yes *versus* No	ALS	0.32 (0.16-0.67)*
			n=1.028 *versus* 279	CPR	0.21 (0.06-0.70)*
				MV	0.32 (0.14-0.70)*
Halpern et al.^(28)^	Retrospective	1,121 patients with cancer, at ICU	LW *versus* DPA *versus*	CPR	2.8 *versus* 6.2 *versus* 5.8%
			No LW/DPA	Reduced ALS	9.1 *versus* 8.4 *versus* 6.8%
				MV	61.9 *versus* 58.6 *versus* 56.22%
			n=176 *versus* 534 *versus* 411	Vasopressors	42.6 *versus* 46.6 *versus* 43.8%
				Hemodialysis	10.8 *versus* 9.2 *versus* 10%
Garrido et al.^(29)^	Prospective	336 patients with advanced cancer	LW/DPA *versus*	Quality of life	QoL: 6.4 *versus* 6.2 p:0.49
			No LW/DPA	Costs	Costs: p:0.49
			n=178 *versus* 158		QoL: 6.7 *versus* 6.0 p:0.01*
			DNR *versus* No DNR		Costs: p:0.12
			n=136 *versus* 195		

* p<0.05. AD: Advanced Directives; aOR: adjusted odds ratio; ICU: intensive care unit; CPR: cardiopulmonary resuscitation; DNR: do not resuscitate; MV: mechanical ventilation; LW: living will; DPA: durable power of attorney ; ALS: advanced life support.

The majority of studies (6; 86%) showed that the existence of some type of AD accounted for lower rates of invasive interventions in the last days of patient´s life, including length of hospital stay, admission to intensive care unit (ICU), death at home, CPR maneuvers, use of vasoactive drugs, mechanical ventilation, artificial nutrition, hemodialysis, among others.

Silveira et al., interviewed the caregivers of 3,746 elderly Americans aged over 60 years, who died between 2000 and 2006, in order to evaluate how many of these patients who had lost their decision-making ability (n=999) had their preferences respected in their directives. Disabled patients who had AD were less likely to receive any possible treatment (adjusted *odds ratio* (aOR=0.33; 95% confidence interval - 95%CI: 0.19 - 0.56), had more limited treatment (aOR=1.79; 95%CI: 1.28-2.50), and more comfort (aOR=2.59; 95%CI: 1.06-6.31), as well as a tendency to reduce death at hospital (aOR=0.71; 95%CI: 0.47-1.07) than those who did not have AD.^8^

In a retrospective study of 422 American patients who died after allogeneic bone marrow transplantation from 2008 to 2015, the authors found that those with AD were less likely to be admitted to ICU (41% *versus* 52%; p=0.03), be on mechanical ventilation (21% *versus* 37%; p<0.01), and die at ICU ( *odds ratio* - OR=0.44; 95%CI: 0.27-0.72) than patients without directives.^26^ With similar findings, a prospective study in Taiwan included 1,307 elderly over 65 years and with chronic diseases. Patients with directives were less likely to receive ALS.^27^

On the other hand, Halpern et al., evaluated cancer patients admitted to ICU between 2006 and 2008 and found there were no differences regarding the procedures and therapies conducted during the ICU stay, as well as in length of hospital stay and place of death between patients with or without directives (LW or DPA).^28^ Similar data were found by Hartog et al., when studying patients who died at the ICU of a German hospital.^7^ However, unlike Halpern et al., they observed that patients with AD had more DNR (77% *versus* 56%; p=0.007), and lower probability of CPR (9% *versus* 23%, p=0.029) than patients without directives.^28^

The association between different types of directives or medical orders (LW, DPA, and DNR), quality of life, and care costs was the endpoint of an American study with 336 patients with advanced cancer. The presence of DNR in the whole sample was associated with better quality of life (p=0.01), but this was not seen among patients with LW or DPA (p=0.49). The study was not able to demonstrate cost differences between having or not directives (DNR, p=0.12, and LW/DPA, p=0.31).^29^

In a systematic review of 45 observational studies evaluating the effects of AD (LW/DPA) on end-of-life care, there was a reduction in the use of ALS in 10 of 22 studies; reduction of death at hospital in 2 of 6 studies; increased use of hospice or palliative care in 5 of 7 studies; absence of benefit in quality of life in all 6 studies, but with a reduction in symptoms of patients and relatives in 3 of 8 studies.^9^

## DISCUSSION

In this narrative review of the variables that influence the medical decision regarding AD and their impact on end-of-life care, we found that knowledge and experience with AD are associated with greater acceptance of patient´s choices. Research has shown that physicians who study more on this subject, work with chronic and terminally ill patients and with palliative care, have a higher compliance to AD.^9 , 14 , 22^

The main factors related to healthcare professionals not implementing the directives were the clinical context of patients, medical paternalism and patients not understanding their true clinical condition. Most physicians will not implement a directive if the patient’s clinical condition is reversible with medical treatment. In addition, Schaden et al., demonstrated that the level of compliance to AD depends on each topic present in it. For example, in relation to CPR, the agreement rate with AD was 99%; nevertheless, when it comes to the use of mechanical ventilation or nutrition, the agreement rate was 80% and 78%, respectively.^30^ Likewise, physicians believe they know what is best for their patients and that all effort must be made to treat them.^19^ However, the best for the patient in the medical opinion may not be the same in the patient view.^18^ In addition, most directives are vague and do not specify exactly which treatments and procedures are allowed or not. It is worth noting the medical concern about the patient fully understanding a directive and its consequences, since the directives often do not address the patients’ real desires.^6^

We have shown that legal issues, family influences and cultural factors contribute to a lesser extent to not implementing the directives. However, in countries where there is less knowledge about its use, lack of clear legislation on the subject, family pressure, and the cultural values of the region, the medical decision may be contrary.

Although the literature presents contradictory results, we demonstrated that having directives somehow contributes to less invasive measures in terminally ill individuals. In our review, the only negative study in all its aspects was carried out by Halpern et al., We highlight, in that case, the low rate of LW, with a higher prevalence of exclusive DPA. Often family members or legal guardians are not able to express patients’ preferences, in addition to being emotionally involved in the situation.^28^ Admission to intensive care units often cause patients or their families wish to have some kind of treatment. This corroborates the findings by Hartog et al., who, in the ICU scenario, did not find a reduction in the use of mechanical ventilation, circulatory support and hemodialysis.^7^

In the systematic review included, the data are mixed and diverse, but suggest that having directives may contribute to reduced therapeutic obstinacy, without presenting an essential impact on end-of-life care and patient satisfaction.^9^

Our study has several limitations. The most important is that it is a narrative review, and the studies analyzed were randomly chosen by the authors. Selected studies used diverse methods (qualitative, quantitative, mixed, prospective and retrospective) and different primary endpoints. Some presented the attitude of physicians towards the directives, others the decision-making process at the end of life, in such a way that the extraction and interpretation of the data could be biased. We emphasize the selection of studies published in English, Portuguese and Spanish, eventually excluding relevant publications in other languages, which could have enriched the review. In addition, the different periods analyzed ranged from 1997 to 2018, that is, more than 20 years. During this interval, the attitude of physicians to the directives could have changed over time, with changes in existing laws and greater recognition of the importance of palliative care in the health care.

Regarding the impact of directives on end-of-life care, it is important to make clear that the majority of studies was observational. They also considered different populations (cancer patients, ICU patients, with advanced dementia, transplanted, and with chronic diseases), in diverse settings, (ICU, hospitals, and hospices), with distinct endpoints.

## CONCLUSION

Most physicians were interested in respecting the autonomy of their patients and agreed that having advanced directives helps in the decision-making process; however, they mentioned the existence of other factors that were taken into account. The most important factors for the medical decision are patients’ prognosis and conditions of reversibility. Other aspects mentioned were knowledge and experience of physicians with the use of directives, legal concerns, family influences, cultural and religious factors. Having directives contributed to reduced employment of life-sustaining treatments and increased adoption of comfort measures.

Although advanced directives have increased worldwide, they are still unsatisfactory. Strategies should be used to increase their implementation, especially in regions where this issue has not been explored yet. It is paramount that physicians, especially those dealing with chronic or critically ill patients, discuss and guide their patients on the existence of directives as well as on the entire formalization process. For this to occur more quickly and efficiently, in addition to the continuing education of healthcare professionals, it is necessary to advance in the regulation of Advance Directives, primarily in countries where they have not been legalized yet.
