# Supplementary material for: Variables that influence the medical decision regarding Advance Directives and their impact on end-of-life care
Source: Einstein (Sao Paulo). 2019 Oct 3;18:eRW4852. doi: 10.31744/einstein_journal/2020RW4852 (PMC6896595; doi:10.31744/einstein_journal/2020RW4852)
Supplement: Translation

# Translation: Variáveis que influenciam na decisão médica frente a uma Diretiva Antecipada de Vontade e seu impacto nos cuidados de fim de vida

**Authors:** Larissa Mont’Alverne de Arruda, Kelline Paiva Bringel Abreu, Laryssa Braga Cavalcante Santana, Manuela Vasconcelos de Castro Sales

**Affiliations:** 1 Brasil Hospital Haroldo Juaçaba, Instituto do Câncer do Ceará, Fortaleza, CE, Brasil.; 2 Brasil Instituição Federal das Unimeds do Estado do Ceará, Fortaleza, CE, Brasil.; 3 Brasil Hospital de Messejana Dr. Carlos Alberto Studart Gomes, Fortaleza, CE, Brasil.; 4 Brasil Hospital Universitário Walter Cantídio, Fortaleza, CE, Brasil.

**Keywords:** Diretivas antecipadas, Testamentos quanto à vida, Médicos, Atitude, Tomada de decisões, Planejamento antecipado de cuidados

## Abstract

O objetivo deste estudo foi identificar as variáveis que influenciam na aderência dos médicos às Diretivas Antecipadas de Vontade e avaliar seu impacto nos cuidados de fim de vida. Trata-se de revisão narrativa de literatura, com levantamento de 25 artigos publicados nos bancos de dados Capes, EBSCOhost, BDTD, BVS, Google Scholar, MEDLINE^®^/PubMed, no período de 1997 a 2018. Os descritores de saúde utilizados foram: “diretivas antecipadas”, “testamentos quanto à vida”, “médicos”, “atitude”, “tomada de decisões” e “planejamento antecipado de cuidados”. Os principais fatores que influenciaram os médicos na aderência às diretivas foram prognóstico do paciente, paternalismo médico, e entendimento do paciente sobre sua condição clínica. Respeito à autonomia, falta de conhecimento e experiência no uso de diretivas, preocupações legais, influência de familiares, fatores culturais e religiosos também contribuíram para a decisão médica. A maioria dos estudos (86%) evidenciou que a presença de uma diretiva foi responsável por menores taxas de intervenções invasivas nos últimos dias de vida dos pacientes. Médicos apresentaram interesse em respeitar a autonomia de seus pacientes e concordaram que a existência de uma diretiva avançada auxilia no processo de tomada de decisão, porém afirmam que outros fatores são levados em consideração, principalmente o prognóstico do paciente e as condições de reversibilidade. A presença de diretivas contribuiu para a redução do uso de terapias de suporte de vida e adoção de medidas de conforto.

## INTRODUÇÃO

Os avanços tecnológicos contínuos fazem com que os profissionais busquem prolongar a vida de seus pacientes, independente das condições em que estejam vivendo. Na terminalidade, as medidas de suporte invasivo já não aumentam a sobrevida do paciente, apenas prolongam seu processo de morte. A medicina atual vive um momento de busca pelo sensato equilíbrio na relação médico-paciente, visando garantir sua autonomia, como o princípio da não maleficência. Dessa forma, as Diretivas Antecipadas de Vontade (DAV) vêm ganhando destaque nas definições do plano de cuidado do paciente.^( 1 , 2 )^

As DAV são declarações escritas antecipadamente por pacientes, permitindo que eles manifestem prévia e expressamente seus desejos e preferências de tratamento, de forma livre e autônoma, em momentos finais de sua existência, em virtude de doenças ou incapacidades permanentes. Visam proteger a autonomia do paciente, caso ele se torne incapacitado no futuro para decidir por si próprio.^( 3 - 6 )^

Estudos evidenciam que a maioria de pacientes críticos perde a capacidade de tomada de decisão, sendo, então, esta assumida cada vez mais de forma complexa por seus familiares e médicos assistentes. É necessário que os desejos dos pacientes frente a procedimentos e tratamentos futuros sejam detectados previamente.^( 7 , 8 )^Apesar da maioria dos médicos demonstrar atitudes positivas em relação às DAV, nem sempre a equipe assistente segue o planejamento de cuidado determinado na DAV.^( 5 , 9 , 10 )^

Objetivamos, neste estudo, revisar a literatura para identificar as variáveis que influenciam na aderência dos médicos às DAV e avaliar o impacto da presença das DAV nos cuidados de fim de vida.

Este estudo constitui-se de uma revisão narrativa de literatura realizada entre julho e outubro de 2018, na qual se efetuou levantamento dos artigos publicados nos últimos 21 anos (1997 a 2018) em inglês, espanhol e português, nos bancos de dados da Coordenação de Aperfeiçoamento de Pessoal de Nível Superior (CAPES), EBSCOhost, Biblioteca Digital Brasileira de Teses e Dissertações (BDTD), Biblioteca Virtual em Saúde (BVS), Google Scholar e MEDLINE^®^/PubMed. Foram utilizados os Descritores em Ciências da Saúde: “ *advance directives* ”; “ *living wills* ”; “ *physicians* ”; “ *attitude* ”; “ *decision making* ”; “ *advance care planning* ”, além dos operadores booleanos E/OU ( *AND/OR* ).

Consideramos todos os artigos que identificassem qualquer um dos seguintes fatores: a atitude dos médicos frente às diretivas (positiva, negativa ou mista) com suas variáveis no processo de decisão e/ou os efeitos da presença das diretivas nos cuidados de fim de vida. Foram incluídos artigos originais (estudos observacionais, estudos de coorte, ensaios clínicos) e revisões sistemáticas com metodologia quantitativa, qualitativa ou mista. Foram excluídos publicações em teses, capítulos de livros, relatos de casos, publicações em outros idiomas que não português, espanhol e inglês, estudos que identificassem apenas a atitude de profissionais de saúde não médicos, artigos com ênfase apenas no conhecimento e na experiência dos médicos frente às diretivas e artigos duplicados.

Definimos como diretivas a presença de manifestação explícita da própria vontade ou “ *living will* ” (LW), poder permanente do responsável legal (DPA - *durable power of attorney* ), ambos (LW e DPA), ordem médica de não reanimar (ONR).

### Variáveis que influenciam na aderência dos médicos às Diretivas Antecipadas de Vontade

Durante o processo de busca no banco de dados, foram selecionados, incialmente, 88 artigos, por meio da leitura de seus resumos. Após análise detalhada dos critérios de inclusão e exclusão empregados, além dos artigos encontrados em duplicata (apenas dois), foram excluídos 63 estudos. Dos 25 estudos incluídos (1 revisão sistemática e 24 artigos originais), as variáveis que influenciam na aderência dos médicos às diretivas foram analisadas em 18 estudos ( Tabela 1 ).

**Tabela 1:** Fatores que influenciam a atitude dos médicos frente às Diretivas Avançadas de Vontade

Estudos	Autonomia	Conhecimento e experiência	Cenário específico	Paternalismo e compreensão dos pacientes	Questões legais	Familiar	Fatores culturais e religiosos
Rossini et al.^(1)^		+					
Quantitativo							
Questionário							
Sittisombut et al.^(2)^							+
Quantitativo							
Questionário							
Bradley et al.^(6)^				+			
Qualitativo							
Entrevista							
Hildén et al.^(11)^	+						
Quantitativo							
Questionário							
Torke et al.^(12)^	+		+				
Quantitativo							
Questionário							
Thompson et al.^(13)^	+						
Qualitativo							
Entrevista							
Navarro et al.^(14)^		+					
Quantitativo							
Questionário							
Velasco-Sanz et al.^(15)^		+					
Quantitativo							
Questionário							
Peicius et al.^(16)^		+					
Quantitativo							
Questionário							
Burkle et al.^(17)^			+		+	+	
Quantitativo							
Cenários hipotéticos							
Bond et al.^(18)^				+			
Qualitativo							
Entrevista							
Bentur et al.^(19)^				+			+
Qualitativo							
Entrevista							
Asai et al.^(20)^					+	+	+
Qualitativo							
Entrevista							
White et al.^(21)^					+		
Quantitativo							
Questionário							
Forte et al.^(22)^					+		
Quantitativo							
Questionário							
Escher et al.^(23)^						+	
Quantitativo							
Questionário							
Cenários hipotéticos							
Horn et al.^(24)^							+
Qualitativo							
Entrevista							
Sprung et al.^(25)^							+
Prospectivo							

A partir dos estudos expostos na tabela 1 , os principais fatores que influenciaram os profissionais de saúde na aderência às DAV foram: respeito à autonomia,^( 11 - 13 )^falta de conhecimento médico e experiência no uso de diretivas,^( 1 , 14 - 16 )^paternalismo médico e dificuldades na definição do prognóstico do paciente,^( 6 , 17 - 19 )^preocupações legais,^( 17 , 20 - 22 )^influência de familiares,^( 17 , 20 , 23 )^e fatores culturais e religiosos.^( 2 , 19 , 20 , 24 , 25 )^

O respeito à autonomia foi evidenciado por Torke et al., de modo que 96,6% e 81,8% dos médicos consideraram respectivamente, “respeito do paciente como pessoa” e “o que os pacientes gostariam que tivessem sido feito com eles”, fatores extremamente ou muito importantes na decisão médica.^( 12 )^

O conhecimento e a experiência dos médicos no uso de diretivas estão relacionados diretamente com seu maior emprego na tomada de decisão.^( 1 , 14 - 16 )^A maioria concorda que a presença de diretivas e a formalização do documento auxiliam no processo de tomada de decisão. Em estudo com médicos de família, aproximadamente 70% tinham conhecimento sobre o tema, porém a ausência de um maior entendimento específico para o aconselhamento de seus pacientes foi uma de suas principais barreiras.^( 14 )^Outros estudos realizados em países em que as DAV ainda não são legalizadas revelam taxas de desconhecimento bem maiores, variando de 50 a 74%.^( 1 , 16 )^

Burkle et al., sugeriram que a aderência médica a uma diretiva é “situação especifica”, e o julgamento médico é mais importante do que seguir uma DAV existente, dependendo da condição clínica do paciente. A concordância seria maior nas situações de doenças crônicas, de terminalidade ou de sofrimento do paciente, ao passo que, nas de emergência e/ou reversíveis, prevaleceria o julgamento médico.^( 17 )^

O paternalismo de profissionais de saúde, ou seja, a decisão de uma intervenção terapêutica médica sem considerar a opinião do paciente buscando seu benefício e a falta de compreensão deste sobre a real condição de sua enfermidade também interferem neste processo de tomada de decisão médica.^( 6 , 18 , 19 )^Bond et al., demonstraram que o nível de concordância com as diretivas está diretamente relacionado com o que os médicos julgam ser melhor para o doente, sugerindo que se ele dispusesse de todo o conhecimento científico, concordaria com a decisão médica.^( 18 )^Estudos sugerem que os pacientes não são informados corretamente durante a realização de uma diretiva, de tal forma que esta pode representar uma má interpretação de seus desejos.^( 6 )^

Em países onde as DAV são amparadas legalmente muito se questiona sobre a influência legal na tomada de decisão médica.^( 17 , 20 - 22 )^Em pesquisa realizada em três Estados diferentes na Austrália, com suas respectivas leis, a maioria dos entrevistados considerou as DAV no processo de decisão, porém outros fatores foram mais importantes, como a qualidade de vida do paciente após tratamento proposto e indicação de tratamento clínico, ao invés da obrigação legal de contemplar uma diretiva.^( 21 )^Muitos médicos, entretanto, admitem que o temor por responsabilizações legais pode afetar suas condutas diante de um paciente. Em cenários hipotéticos de discordância entre opiniões familiares e de uma diretiva existente, 53% dos entrevistados consideraram as preocupações legais importantes ou muito importantes para sua decisão.^( 17 )^Da mesma forma, Forte et al., evidenciaram que 44% dos médicos alterariam sua conduta, se não fosse pelo receio de processos legais e da opinião da sociedade.^( 22 )^

Influências familiares também são apontadas como razões para atitudes negativas de médicos frente às escolhas do paciente.^( 17 , 20 , 23 )^Burkle et al., constataram que 75% dos médicos honrarão a diretiva de uma paciente, independente da opinião de seu familiar. Porém, dentre os que não a cumpriram, a maioria (77%) considerou a opinião do familiar um fator importante para sua decisão.^( 17 )^

Outro aspecto negativo na implantação das DAV são fatores culturais e religiosos.^( 2 , 19 , 20 , 24 , 25 )^Médicos asiáticos tendem a discutir prognóstico de seus pacientes com familiares e, dessa forma, podem não respeitar diretivas presentes.^( 2 , 20 )^Sittisombut et al., evidenciaram que 61,8% dos médicos tailandeses nunca questionaram os pacientes com doença terminal se desejavam ser submetidos a manobras de ressuscitação cardiopulmonar (RCP), porém, 94,5%, discutiram esse assunto com os familiares.^( 2 )^Sprung et al., afirmaram que médicos protestantes, católicos ou sem afiliação religiosa interrompiam terapias de Suporte Avançado de Vida (SAV) mais frequentemente do que gregos ortodoxos, judeus ou muçulmanos.^( 25 )^

### Impacto da presença das Diretivas Antecipadas de Vontade nos cuidados de fim de vida

O impacto da presença das diretivas nos cuidados de fim de vida foi avaliado em sete estudos ( Tabela 2 ).^( 7 - 9 , 26 - 29 )^

**Tabela 2:** Influência das Diretivas Antecipadas de Vontade nos cuidados de fim de vida

Estudo	Tipo	População	DAV	Desfecho	AOR (IC95%)
Hartog et al.^(7)^	Retrospectivo	192 pacientes da UTI	Sim *versus* Nãon=64 *versus* 128	RCP	9,4 *versus* 22,8%*
				ONR	76,6 *versus* 56,3%*
				VM	79,7 *versus* 89,7%
				Vasopressores	85,9 *versus* 87,9%
				Hemodiálise	42,2 *versus* 46,3%
Silveira et al.^(8)^	Retrospectivo	999 pacientes > 60 anos	LW *versus* Não LW	Morte Hospitalar	0,71 (0,47-1,07)
			n=444 *versus* 552	Todo	0,33 (0,19-0,56)*
			DPA *versus* Não DPA	Limitado	1,79 (1,28-2,50)*
			n=589 *versus* 407	Conforto	2,59 (1,06-6,31)*
				Morte hospitalar	0,72 (0,55-0,93)*
				Todo	0,54 (0,34-0,86)*
				Limitado	1,18 (0,75-1,85)
				Conforto	2,01 (0,89-4,52)
Brinkman-Stoppelenburg et al.^(9)^	Revisão sistemática	113 pacientes com câncer, demência avançada, trauma ou cardiopatia	n=45 Sim *versus* Não	SAV (n=22)	10/22 estudos positivos
				Qualidade de vida (n=6)	0/6 estudos positivos
				Tempo internamento (n=8)	2/8 estudos positivos
				Cuidados paliativos (n=7)	5/7 estudos positivos
				Estresse familiar/paciente (n=8)	3/8 estudos positivos
				Concordância com os desejos dos pacientes (n=2)	0/2 estudos positivos
Cappell et al.^(26)^	Retrospectivo	422 pacientes submetidos a transplante alogênico	Sim *versus* Não	Internamento em UTI	41 *versus* 52%*
			n=184 *versus* 238	VM	21 *versus* 37%*
				Óbito na UTI	0,44 (0,27-0,72)*
Yen et al.^(27)^	Prospectivo	1.307 pacientes > 65 anos portadores de doenças crônicas	Sim *versus* Não	SAV	0,32 (0,16-0,67)*
			n=1.028 *versus* 279	RCP	0,21 (0,06-0,70)*
				VM	0,32 (0,14-0,70)*
Halpern et al.^(28)^	Retrospectivo	1.121 pacientes com câncer na UTI	LW *versus* DPA *versus*	RCP	2,8 *versus* 6,2 *versus* 5,8%
			Não LW/DPA	VM	9,1 *versus* 8,4 *versus* 6,8%
				Redução SAV	61,9 *versus* 58,6 *versus* 56,22%
			n=176 *versus* 534 *versus* 411	Vasopressores	42,6 *versus* 46,6 *versus* 43,8%
				Hemodiálise	10,8 *versus* 9,2 *versus* 10%
Garrido et al.^(29)^	Prospectivo	336 pacientes com câncer avançado	LW/DPA *versus*	Qualidade de vida	QoL: 6,4 *versus* 6,2 p:0,49
			Não LW/DPA	Custos	Custos: p:0,49
			n=178 *versus* 158		QoL: 6,7 *versus* 6,0 p:0,01*
			ONR *versus* Não ONR		Custos: p:0,12
			n=136 *versus* 195		

* p<0,05. DAV: Diretivas Antecipadas de Vontade; AOR: *odds ratio* ajustada; UTI: unidade de terapia intensiva; RCP: ressuscitação cardiopulmonar; ONR: ordem médica de não reanimar; VM: ventilação mecânica; LW: *living will* ; DPA: poder permanente do responsável legal; SAV: suporte avançado de vida.

A maioria dos estudos evidenciou (6; 86%) que a presença de algum tipo de DAV foi responsável por menores índices de intervenções invasivas nos últimos dias de vida dos pacientes, desde o tempo de permanência hospitalar, internamento em unidade de terapia intensiva (UTI), morte no domicílio, uso de manobras de RCP, drogas vasoativas, ventilação mecânica, nutrição artificial, hemodiálise, além de outros.

Silveira et al., entrevistaram os cuidadores de 3.746 idosos americanos acima de 60 anos, que faleceram no período de 2000 a 2006, com o objetivo de avaliar quantos destes pacientes que perderam a capacidade de decisão (n=999) tiveram suas preferências respeitadas em suas diretivas. Pacientes incapazes que possuíam DAV foram menos propensos a receber todo tratamento possível ( *odds ratio* ajustada − AOR=0,33; intervalo de confiança de 95% − IC95%: 0,19-0,56), mais tratamentos limitados (AOR=1,79; IC95%: 1,28-2,50) e de conforto (AOR=2,59; IC95%: 1,06-6,31), além de tendência de redução de morte hospitalar (AOR=0,71; IC95%: 0,47-1,07) do que aqueles que não apresentavam DAV.^( 8 )^

Em estudo retrospectivo de 422 pacientes americanos falecidos após realização de transplante alogênico de medula, durante o período de 2008 a 2015, os autores evidenciaram que aqueles com DAV estavam menos propensos a internamentos em UTI (41% *versus* 52%; p=0,03), ao uso de ventilação mecânica (21% *versus* 37%; p<0,01) e a óbito em UTI ( *odds ratio* − OR=0,44; IC95%: 0,27-0,72) do que pacientes sem diretivas.^( 26 )^Com achados semelhantes, estudo prospectivo realizado em Taiwan incluiu 1.307 idosos acima de 65 anos e com doenças crônicas. Pacientes com diretivas apresentaram menor probabilidade de receberem SAV.^( 27 )^

Por outro lado, Halpern et al., avaliando pacientes oncológicos admitidos na UTI entre 2006 e 2008, evidenciaram que não houve diferenças em relação aos procedimentos e terapias realizados durante o internamento na UTI, como também no tempo de internamento e local de óbito dos pacientes com ou sem diretivas (LW ou DPA).^( 28 )^Dados semelhantes foram encontrados por Hartog et al., ao estudarem pacientes falecidos na UTI de um hospital alemão.^( 7 )^Entretanto, diferentemente de Halpern et al., observou-se que pacientes com DAV apresentavam mais ONR (77% *versus* 56%; p=0,007) e menor probabilidade de RCP (9% *versus* 23%; p=0,029) do que pacientes sem diretivas.^( 28 )^

A associação entre diferentes tipos de diretivas ou ordens médicas (LW, DPA e ONR), qualidade de vida e custos de cuidado foi o desfecho de um estudo americano com 336 pacientes com câncer avançado. A presença de ONR em toda a amostra foi associada a uma melhor qualidade de vida (p=0,01), porém isto não foi observado entre os pacientes que apresentavam LW ou DPA (p=0,49). O estudo não conseguiu demonstrar diferenças de custos entre a presença de diretivas ou não (ONR, p=0,12, e LW/DPA, p=0,31).^( 29 )^

Em revisão sistemática de 45 estudos observacionais que avaliavam os efeitos das DAV (LW/DPA) nos cuidados de fim de vida, observaram-se: redução do uso de SAV em 10 de 22 estudos; redução de morte hospitalar em 2 de 6 estudos; aumento do uso de *hospice* ou cuidado paliativo em 5 de 7 estudos; ausência de benefício na qualidade de vida em todos os 6 estudos, porém com redução dos sintomas de pacientes e familiares em 3 de 8 estudos.^( 9 )^

## DISCUSSÃO

Nesta revisão narrativa sobre as variáveis que influenciam na decisão médica frente às DAV e o impacto destas nos cuidados de fim de vida, evidenciamos que o conhecimento e a experiência com uso das DAV estão associados a maior aceitação das escolhas do paciente. Pesquisas demonstram que médicos que estudam mais sobre este assunto, trabalham com doentes crônicos e terminais e com cuidados paliativos apresentam maior aderência às DAV.^( 9 , 14 , 22 )^

Os principais fatores relacionados ao não seguimento das diretivas pelos profissionais de saúde foram contexto clínico em que o paciente se encontrava, paternalismo médico e falta de entendimento do paciente de sua real condição clínica. A maioria dos médicos não aderirá a uma diretiva, caso a condição clínica do paciente seja reversível com tratamento médico. Além disso, Schaden et al., demonstraram que o nível de aderência a uma DAV depende de cada tópico presente nela. Por exemplo, em relação a manobras de RCP, a taxa de concordância com a DAV foi de 99%, porém, quando se trata do uso de ventilação mecânica ou nutrição, a taxa de concordância foi de 80% e 78%, respectivamente.^( 30 )^Da mesma forma, médicos acreditam saber o que é melhor para seu paciente e que todo o esforço deve ser realizado para tratá-lo.^( 19 )^Porém, o melhor para o doente na opinião médica, talvez não o seja, na visão do paciente.^( 18 )^Além disso, a maioria das diretivas é vaga e não especifica exatamente quais tratamentos e procedimentos são permitidos ou não. Vale ressaltar a preocupação médica sobre o completo entendimento e as consequências de uma diretiva pelo paciente − muitas vezes as diretivas não contemplam os reais desejos dos pacientes.^( 6 )^

Evidenciamos que questões legais, influências familiares e fatores culturais contribuem em menor proporção para a aderência negativa às diretivas. Porém, em países onde há um desconhecimento maior sobre seu uso, ausência de uma legislação clara sobre o assunto, pressão de familiares, além dos valores culturais da região, a decisão médica pode ser contrária.

Apesar da literatura evidenciar resultados contraditórios, demonstramos que a presença de diretivas contribui, de alguma forma, para a implantação de medidas menos invasivas na terminalidade. Em nossa revisão, o único estudo negativo em todos seus quesitos foi o de Halpern et al. Ressaltamos, neste caso, a baixa taxa de LW, com maior predomínio de DPA exclusivo. Muitas vezes, os familiares ou responsáveis legais não são capazes de exprimir as preferências dos pacientes, além de estarem emocionalmente envolvidos na situação.^( 28 )^Internamentos em centros de suporte intensivo geralmente fazem com que paciente ou seus familiares desejem a realização de algum tipo de tratamento. Isto corrobora os achados evidenciados por Hartog et al., que, no cenário de UTI, não apresentaram redução do uso de ventilação mecânica, suporte circulatório e hemodiálise.^( 7 )^

Na revisão sistemática incluída, os dados são mistos e diversos, porém sugerem que a presença de diretivas pode contribuir para redução da obstinação terapêutica, sem apresentar impacto essencial no cuidado de fim de vida e satisfação do paciente.^( 9 )^

Nosso estudo apresenta várias limitações. A mais importante é que se trata de uma revisão narrativa, e os estudos analisados foram escolhidos aleatoriamente pelos autores. Estudos selecionados utilizaram metodologias diversas (qualitativos, quantitativos, mistos, prospectivos e retrospectivos) e diferentes desfechos primários. Alguns apresentavam a atitude dos médicos frente às diretivas, outros o processo de decisão no fim de vida, de tal forma que a extração e interpretação dos dados poderia contribuir com viés. Ressaltamos a seleção de estudos publicados em inglês, português e espanhol, excluindo possíveis publicações importantes em outros idiomas, que poderiam ter enriquecido a revisão. Além disso, o período empregado em diferentes épocas foi de 1997 a 2018, ou seja, mais de 20 anos. Durante este período, a atitude dos médicos frente às diretivas poderia se alterar ao longo do tempo, com a mudança de legislações vigentes e o maior reconhecimento da importância dos cuidados paliativos na área da saúde.

Em relação ao impacto das diretivas sobre os cuidados de fim de vida, importante esclarecermos que a maioria dos estudos foi de caráter observacional. Também considerou populações diferentes (pacientes oncológicos, em UTI, com demência avançada, transplantados e com doenças crônicas), em cenários diferentes (UTI, hospital e casas de repouso), com desfechos distintos.

## CONCLUSÃO

A maioria dos médicos apresenta interesse em respeitar a autonomia de seus pacientes e concorda que a presença de uma diretiva antecipada de vontade auxilia no processo de tomada de decisão, porém afirma existência de outros fatores que são levados em consideração. Os fatores mais importantes para a decisão médica são o prognóstico do paciente e as condições de reversibilidade. Outros aspectos citados foram conhecimento e experiência dos médicos com uso de diretivas, preocupações legais, influências familiares, fatores culturais e religiosos. A presença de diretivas contribuiu para a redução de Suporte Avançado de Vida e adoção de medidas de conforto.

Apesar das taxas de conclusão das diretivas aumentarem em todo o mundo, ainda são insatisfatórias. Estratégias devem ser utilizadas para elevar sua implantação, principalmente nas regiões onde este assunto ainda não é tão explorado. É de fundamental importância que os médicos, principalmente os que lidam com doentes crônicos ou críticos, discutam e orientem seus pacientes quanto à existência das diretivas como também todo seu processo de formalização. Para que isto ocorra de forma mais rápida e eficiente, além da educação continuada dos profissionais de saúde, faz-se necessário avançar na regulamentação das Diretivas Antecipadas de Vontade, principalmente nos países onde ainda não são legalizadas.
